# The relationship between right ventricular functions and early clinical events during hospitalization and after discharge in patients with low ejection fraction heart failure

**DOI:** 10.3389/fcvm.2025.1584894

**Published:** 2025-06-19

**Authors:** Mehdi Onac, Ali Yasar Kılınc, Ahmad Huraibat, Alaa R. AL-Ihribar, Umuttan Dogan

**Affiliations:** ^1^ Department of Cardiology, İstanbul Aydın University Medical Faculty Hospital, İstanbul, Türkiye; ^2^ Cardiology Department, Akdeniz University Medical Faculty Hospital, Antalya, Türkiye; ^3^ Faculty of Medicine, İstanbul Aydin University, İstanbul, Türkiye; ^4^ Cardiology Department, Medical Park, İstanbul, Türkiye; ^5^ Collage of Medicine and Health Sciences, Palestine Polytechnic University, Hebron, Palestine

**Keywords:** left heart failure, right ventricle strain, diastolic dysfunction, tissue Doppler echocardiography, longitudinal 2D strain analysis, speckle tracking the relationship between right ventricular functions and early clinical events during hospitalization and after discharge in patients with low ejection fraction heart failure

## Abstract

**Background and aim:**

Right ventricular dysfunction is an independent predictor of poor prognosis in the patients with left ventricle failure. In this study, it is aimed to investigate the relationship between RV functions and early clinical events in hospital and after discharge in patients who were followed up for heart failure with a low ejection fraction and were hospitalized with the diagnosis of heart failure.

**Materials and methods:**

Seventy patients with a left ventricular ejection fraction below 45% who were hospitalized due to decompensated heart failure were enrolled in this study. They were stabilized after medical treatment and discharged. Thereafter, they were followed up for 6 months in terms of clinical events. Image windows used as standard in the 2D echocardiography technique were recorded while the related technique was being performed.

**Results:**

Composite adverse events were observed in 45 patients (64.3%), while composite adverse events were not observed in 25 patients (35.7%). Relation between the independent risk factors and composite adverse events were analyzed with binary logistic regression analysis. Receiver Operating Characteristic (ROC) analysis was performed to determine the distinctive performance of right ventricular (RV) Global Longitudinal Strain (GLS) and RV Free Wall Strain (FWS) parameters in the prediction of mortality in patients. RV Global LS (*p* < 0.001) and RV Free W Strain (*p* < 0.001) were determined as distinguishing factors related with mortality.

**Conclusion:**

We found that RV GLS and RV fwLS are closely related. Moreover, both measurements were correlated not only with parameters reflecting RV systolic function, but also with parameters reflecting LV function.

## Introduction

Heart failure (HF) can be defined as a structural or functional disorder related to the heart that leads to the inability to provide enough oxygen to meet the metabolic needs of the tissues, despite normal filling pressures. Pathological changes in the structure and functions of the left ventricle (LV) over time lead to reshaping in the right ventricle (RV) and result in structural and functional impairment, leading to RV insufficiency. It is thought that the deterioration in the functions of RV has no effect on clinical outcomes, hemodynamic parameters, and overall results in heart patients.The main reason for this is the limitations in imaging methods used to evaluate RV structure and functions.

The anatomical location and complex structure of the RV create limitations in the accurate assessment of reshaping with standard echocardiographic methods. The gold standard diagnostic method for evaluating RV functions is cardiac magnetic resonance imaging; however, its routine use in daily practice is difficult (1, 2). Despite the current limitations, it has been stated that the degree of impairment in RV structure and functions is indicative of cardiovascular endpoints in small-scale studies conducted using relevant methods (3).

In our study, we planned to investigate the relationship between RV functions and early clinical events within the hospital and after discharge in patients with low ejection fraction heart failure (LEFHF) and decompensated heart failure (DHF) who were hospitalized. For RV functions, we preferred to use Tricuspid Annular Plane Systolic Excursion (TAPSE), RV Fractional Area Change (RV FAC), along with parameters of Doppler flow velocities, as well as speckle tracking echocardiography method for RV free wall and global longitudinal shortening-thickening functions. While previous studies have identified the prognostic value of RV function, our study uniquely evaluates the predictive value of RV GLS and RV FWS in a cohort of patients with decompensated low EF heart failure, offering practical metrics that are readily accessible with standard echocardiographic tools.

## Materials and methods

Our study followed 70 patients who were above 18 years of age, receiving HF treatment for at least 3 months with LVEF below 45% and hospitalized with LEFHF for 6 months in terms of clinical events.

Exclusion criteria:

• Moderate to severe mitral valve stenosis
• Severe aortic stenosis, severe aortic valve insufficiency
• Multiorgan failure
• Infiltrative, constrictive and hypertrophic cardiomyopathy
• Congenital heart disease
• Acute myocarditis
• Pregnancy and up to 3 months post-partum
• Patients currently undergoing treatment for active cancer
• Sepsis
• Recently admitted to dialysis due to chronic kidney failure or hospitalized with acute kidney failure
• Cirrhosis
• Cor pulmonale, moderate-advanced stage chronic obstructive pulmonary disease (COPD) or respiratory failure.
• Those who have had a heart attack in the last 6 months or have undergone open heart surgery in the last 6 months (due to valve or coronary artery disease)
• Those with an expected lifespan of less than 1 year due to any illness.
• Patients with poor echogenicity were identified.

We selected a threshold of LVEF <45% to capture patients with clinically significant systolic dysfunction, consistent with ranges used in prior RV strain studies. This range allows for the inclusion of a broader population while still reflecting impaired left ventricular function.

Detailed information was provided to the patients and signed consent forms were obtained. Our study was conducted in accordance with the approval of the Local Ethics Committee (Approval number KAEK-152).

The demographic characteristics of the patients, cardiovascular risk factors, comorbidities, New York Heart Association (NYHA) functional class, and medications used were analyzed.

The treatment and follow-up protocols of the patients included in the study were not interfered with, and the image windows commonly used in the standard 2-dimensional echocardiography technique requested during treatment were recorded when the relevant technique was performed. The subsequently obtained records were analyzed using the speckle tracking echocardiography method software program called Q-LAB (Philips) in a computer environment. The patients' LV, RV functions, all heart chambers, all valve functions were evaluated. Parasternal long axis, short axis midventricular level, apical; 2-chamber, 3-chamber, and 4-chamber views were obtained. Images were recorded during the patient holding their breath for three consecutive heartbeats using two-dimensional, M mode, color doppler, CW doppler, and PW doppler. The color doppler frame scanning rate for color flow tissue Doppler imaging (TDI) images was set to 100–140 Hz. The papillary muscle level was measured in M-mode images for the left ventricle diastolic end diameter and left ventricle systolic end diameter.

Endocardial borders were drawn from the images of apical 2, 3, and 4 chambers using the software available in the echocardiography device, and volumes and EF were calculated using the Biplane Simpson's method. In the evaluation of LV diastolic function, E peak velocity, A peak velocity, deceleration time, e' and a' parameters, and S parameter were used. The records were scanned in the end-systolic phase of the endocardial cavity (minimum cavity area). Apical RV oriented records were taken for RV strain and FAC calculations. Images were detected where the RV was the narrowest and widest during systole and diastole. From these images, RV FAC was calculated by drawing endocardial borders. Records were scanned for endocardial cavity in the end-systolic phase for RV strain, and the program automatically calculated the values of global longitudinal strain (GLS) and RV free wall strain (FWS). *A priori* sample size calculation was conducted based on preliminary data suggesting a 30% incidence of adverse events in heart failure patients with RV dysfunction. With an alpha of 0.05 and power of 80%, a minimum sample size of 64 was estimated. We enrolled 70 patients to account for potential data loss. Echocardiographic evaluations were performed using a Philips EPIQ 7 system, and strain analyses were conducted offline using QLAB software version 10.8 (Philips Healthcare, Andover, MA). Inter-observer and intra-observer variability for RV GLS and FWS were assessed in 20 randomly selected patients by two experienced cardiologists. The intraclass correlation coefficients (ICCs) for RV GLS and RV FWS were 0.92 and 0.89 (intra-observer), and 0.88 and 0.85 (inter-observer), respectively, indicating good reproducibility.

Composite endpoint was defined as the occurrence of one or more of the following clinical events during the follow-up period:

• Cardiovascular Death
• Stroke
• Acute kidney failure (50% increase in creatinine levels according to admission and/or the need for hemodialysis)
• Liver failure (3 times increase in liver enzymes according to admission)
• Rehospitalization
• The duration of stay in the hospital

## Statistical analysis

Categorical variables are presented with frequency (*n*) and percentage (%), normally distributed continuous variables with mean ± standard deviation (SD), and non-normally distributed variables with median (IQR: 25–75th percentile) values. The relationship between categorical variables was evaluated using the Pearson chi-squared test and Fisher's Exact test. The assumption of normal distribution was checked with the Shapiro Wilk test.The Mann–Whitney *U* test was used for the non-parametric comparison of the continuous variables of the two groups, and the Independent *t*-test was used for the parametric comparison.In order to predict mortality, ROC (Receiver Operating Characteristic) analysis was conducted to distinguish patients based on RV Global LS and RV Free W Strain values and determine the cutoff point and the analysis results are presented with Area Under the Curve (AUC), cut-off points, sensitivity and specificity values, and 95% confidence intervals. The optimal cut-off points of the parameters have been calculated using the Youden index. Survival curves were created using the Kaplan–Meier method and overall survival rates were compared between groups using the Log-Rank test. Multivariable logistic regression analysis was conducted to determine factors that independently affect composite outcome in patients and the results were presented with Odds Ratio (OR) and 95% confidence intervals. The factors affecting overall survival were examined using univariate and multivariate Cox regression analysis. In single variable analysis, variables with *p* < 0.2 were used to establish a multiple regression model. The results obtained are presented with hazard ratios (HR) and 95% confidence intervals. All analyses were performed using the IBM SPSS 23.0 program (IBM Corp., Armonk, NY) and *p* values less than 0.05 were considered statistically significant.

## Results

The average age of the patients was 60.1 ± 13.4 years. 85.7% (*n* = 60) of the patients were male. The etiology of 22 patients (31.4%) was non-ischemic, while the etiology of 48 patients (68.6%) was ischemic. The median diagnosis duration of the patients was 36 (IQR: 18–60) months.

In 45 patients (64.3%), composite endpoint occurred, while in 25 patients (35.7%), composite endpoint was not observed. In patients with composite endpoint occurrence, the median QRS duration was significantly higher (*p* = 0.026).

In patients who had composite outcome, 95.6% had NYHA score 2, while in patients who did not have composite outcome, 60% had NYHA score 2, and the difference between them was found to be statistically significant (*p* < 0.001). Information about the general characteristics of the patients is shown in Table 1.

**Table 1:** Patients characters.

Variables	All patients (*n*: 70)	Composite outcome		*p*
		No (*n*: 25)	Yes (*n*: 45)	
Age (years)	60.1 ± 13.4	56.36 ± 14.49	62.18 ± 12.44	0.082
Sex				
Female (*n*, %)	10 (14.3)	5 (20)	5 (11.1)	0.477
Male (*n*, %)	60 (85.7)	20 (80)	40 (88.9)	
Etiology				
Non-ischemic (*n*, %)	22 (31.4)	8 (32)	14 (31.1)	0.939
Ischemic (*n*, %)	48 (68.6)	17 (68)	31 (68.9)	
Diagnosis duration (month)	36 (18–60)	24 (12–48)	36 (24–60)	0.062
Systolic blood pressure (mmHg)	117.91 ± 18.68	120.24 ± 22.4	116.62 ± 16.4	0.442
Diastolic blood pressure (mmHg)	71.03 ± 12.64	70.44 ± 11.55	71.36 ± 13.33	0.774
Heart rate (beats/minute)	76 (67–90)	76 (67–84)	76 (67–90)	0.568
Diabetes mellitus (*n*, %)	40 (57.1)	15 (60)	25 (55.6)	0.719
Hypertension (*n*, %)	41 (58.6)	13 (52)	28 (62.2)	0.405
Smoke (*n*, %)	49 (70)	17 (68)	32 (71.1)	0.785
Variables	All patients (*n*: 70)	Composite outcome		*p*
		No (*n*: 25)	Yes (*n*: 25)	
Medication				
*Beta blocker* (*n*, %)	66 (94.3)	23 (92)	43 (95.6)	0.613
*ACE-ARB* (*n*, %)	65 (92.9)	25 (100)	40 (88.9)	0.152
*MRA* (*n*, %)	48 (68.6)	16 (64)	32 (71.1)	0.539
*Ivabradine (n, %)*	12 (17.1)	6 (24)	6 (13.3)	0.325
*Diuretic* (*n*, %)	69 (98.6)	24 (96)	45 (100)	0.357
*Statin* (*n*, %)	50 (71.4)	19 (76)	31 (68.9)	0.528
QRS duration (msn)	112 (102–134)	108 (92–120)	118 (106–134)	**0**.**026**
Pace-ICD-CRT				
No (*n*, %)	47 (67.1)	18 (72)	29 (64.4)	0.914
ICD (*n*, %)	19 (27.1)	6 (24)	13 (28.9)	
CRT (*n*, %)	4 (5.7)	1 (4)	3 (6.7)	
NYHA				
1 (*n*, %)	12 (17.1)	10 (40)	2 (4.4)	**<0**.**001**
2 (*n*, %)	58 (82.9)	15 (60)	43 (95.6)	
BUN (mg/dl)	27.07 ± 10.63	23.72 ± 8.62	28.93 ± 11.26	**0**.**049**
Creatinine (mg/dl)	1.05 (0.9–1.3)	0.97 (0.88–1.1)	1.1 (0.9–1.36)	**0**.**033**
GFR (ml/min)	67 (53–91)	82 (61–97)	64 (50–85)	0.053
Hemoglobin (g/dl)	12.7 ± 2.19	13.27 ± 2.11	12.39 ± 2.18	0.106

Results are presented as mean ± SD, median (IQR) or *n* (%). Independent *t*-test, Mann–Whitney *U* test, Pearson chi-square test, Fisher's exact test.

Bold values indicate statistically significant results (*p* < 0.05).

When comparing echocardiographic findings of patients based on composite outcome status, it was observed that the left atrial diameter was higher in patients with composite outcome (*p* < 0.001), while values of Mitral A (*p* = 0.026), Mitral Septal E' (*p* = 0.023), Mitral Septal A' (*p* = 0.027), Tricuspid Lateral E’ (*p* = 0.016), Tricuspid Lateral A' (*p* = 0.026), Tricuspid Lateral S' (*p* = 0.013), TAPSE (*p* = 0.018), RV FAC (*p* < 0.001), RV Global LS (*p* < 0.001), and RV Free W Strain (*p* < 0.001) were lower. The analysis results related to the echocardiography findings of the patients are shown in Table 2.

**Table 2:** Echocardiographic findings.

Variables	All patients (*n*: 70)	Composite outcome		*p*
		No (*n*: 25)	Yes (*n*: 45)	
Aortic root (mm)	29.93 ± 3.42	29.84 ± 3.67	29.98 ± 3.31	0.873
Left atrium (mm)	48.4 ± 6.84	44.32 ± 5.9	50.67 ± 6.29	**<0**.**001**
LVED (mm)	64.26 ± 6.93	62.16 ± 7.62	65.42 ± 6.3	0.058
LVES (mm)	55.31 ± 7.97	52.84 ± 8.52	56.69 ± 7.39	0.052
IVS (mm)	9.5 (8–11)	9 (8–10)	10 (8–11)	0.624
PW (mm)	10 (9–10)	10 (9–10)	10 (9–10)	0.516
LVEF (%)	28.19 ± 6.85	29.08 ± 7.35	27.69 ± 6.58	0.419
Mitral E (m/s)	1 (0.7–1.2)	0.9 (0.7–1.2)	1 (0.7–1.2)	0.767
Mitral A (m/s)	0.4 (0–0.8)	0.5 (0.4–0.9)	0.4 (0–0.7)	**0**.**026**
Mitral E/A	1.3 (0.75–2)	1.3 (0.75–1.66)	1.2 (0.8–2)	0.801
Systolic PAB (mmHg)	44.5 (39–57)	41 (37–53)	45 (41–58)	0.081
Mitral Lateral E’ (cm/s)	8 (7–11)	8 (7–9)	8 (7–12)	0.252
Mitral Lateral E/E’	11,4 (7,5–15,7)	12 (8,8–15,7)	10 (7,5–15,7)	0.615
Mitral Lateral A'(cm/s)	5 (4–6)	5 (5–6)	5 (4–6)	0.282
Mitral Lateral S’ (cm/s)	6 (5–7)	6 (5–7)	6 (5–7)	0.482
Mitral Septal E’ (cm/s)	5 (4–7)	6 (5–9)	5 (4–6)	**0**.**023**
Mitral Septal E/E'	17,5 (12–25)	15.5 (10–18.5)	20 (12.8–25)	0.085
Mitral Septal A’ (cm/s)	4 (3–5)	4 (4–6)	4 (3–5)	**0**.**027**
Mitral Septal S’ (cm/s)	5 (5–6)	6 (5–7)	5 (5–6)	0.285
Tricuspid Lateral E’ (cm/s)	10 (7–14)	13 (9–16)	8 (7–12)	**0**.**016**
Tricuspid Lateral A’ (cm/s)	8 (6–11)	10 (8–12)	8 (5–10)	**0**.**026**
Tricuspid Lateral S’ (cm/s)	10 (8–12)	11 (10–12)	10 (8–11)	**0**.**013**
TAPSE (mm)	17.27 ± 3.22	18.48 ± 2.83	16.6 ± 3.25	**0**.**018**
RV FAC (%)	32.92 ± 9.15	38.41 ± 9.66	29.87 ± 7.32	**<0**.**001**
RV global LS (%)	14.25 ± 4.9	17.82 ± 4.02	12.26 ± 3.79	**<0**.**001**
RV free W strain (%)	16.74 ± 5.55	21.09 ± 5.17	14.33 ± 4.13	**<0**.**001**

Results are presented as mean ± SD, median (IQR) or *n* (%). Independent *t*-test, Mann–Whitney *U* test.

Bold values indicate statistically significant results (*p* < 0.05).

Cardiovascular death occurred in 13 patients (18.6%),rehospitalization in 45 patients (64.3%), and acute kidney failure (AKF) was determined in 19 patients (27.1%).It was observed that 15 patients (21.4%) died, while the median mortality period was calculated as 88 days (IQR: 38–140). The median total number of hospitalizations due to RV and LV reasons for patients was 1 (IQR: 1–2).

When echocardiographic findings of the patients were evaluated according to mortality, it was observed that the left atrium diameter was higher in patients who died (*p* = 0.006), Mitral Lateral E/E' (*p* = 0.008), RV Global LS (*p* < 0.001), and RV Free W Strain (*p* < 0.001) values were significantly lower.

ROC analysis was performed to determine the distinguishing performance of RV Global LS and RV Free W Strain parameters in predicting mortality in patients, and the results are presented in Table 3.

**Table 3:** The predictive performance of mortality for RV global LS and RV free W strain parameters.

Variables	AUC (%95 CI)	*P*	Cut-off value	Sensitivity (%)	Specificity (%)
RV global LS	0.837 (0.729–0.914)	**<0**.**001**	≤12.7	100	69.09
RV free W Strain	0.816 (0.706–0.899)	**<0**.**001**	≤14.6	86.67	74.55

Bold values indicate statistically significant results (*p* < 0.05).

**Figure 1:**
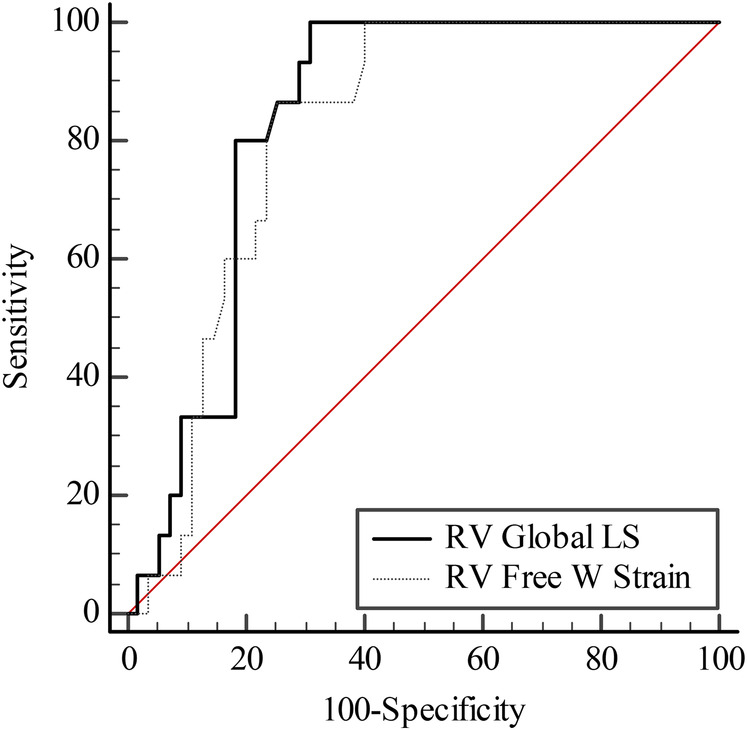
ROC curve for RV global LS and RV free W strain.

It was found that the AUC values obtained in determining mortality of RV Global LS and RV Free W Strain are statistically similar (AUC = 0.837 and AUC = 0.816; *p* = 0.468) (Figure 1).

**Figure 2:**
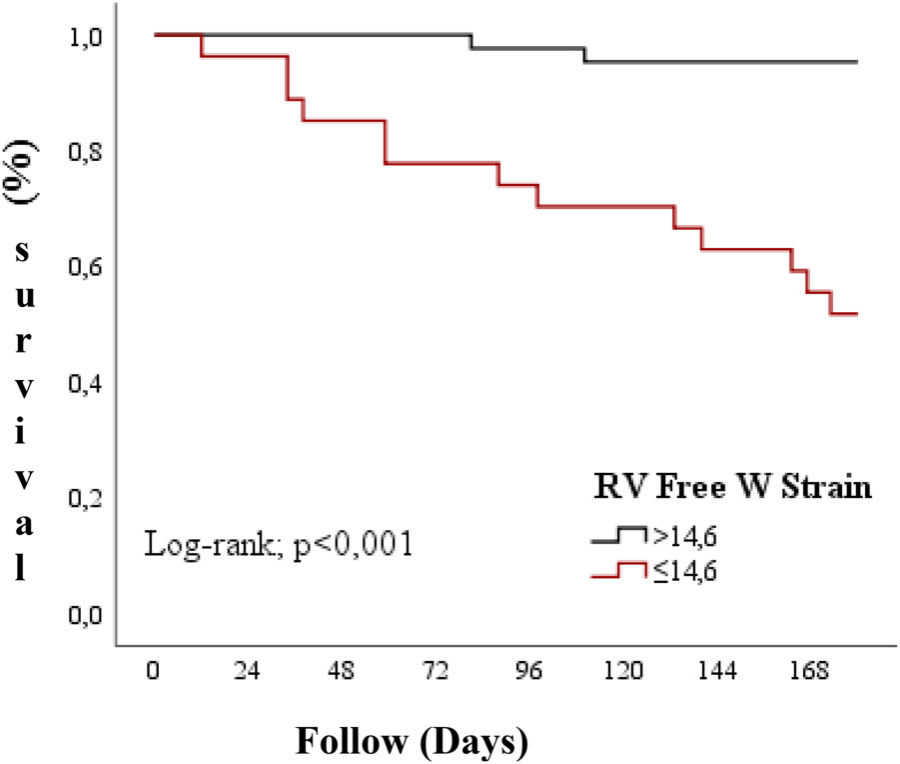
Overall survival according to RV free W strain values.

In patients with RV Free W Strain >14.6, the average survival time was 176.1 days (95% CI: 170.67–179.47), while in those with RV Free W Strain ≤14.6, the average overall survival time was 137.7 days (95% CI: 115.75–159.66).The overall survival rates in the RV Free W Strain ≤14.6 group were found to be significantly lower compared to the RV Free W Strain >14.6 group (Log-rank = 19.239; *p* < 0.001) (Figure 2).

## Discussion

The function of the RV is an important determinant of the progression of heart failure. It has been shown that the LV contraction is responsible for 20%–40% of the RV systolic pressure, and that RV contraction contributes 4%–10% to the LV systolic pressure (4). In daily practice, deformation analysis and parameters derived from this analysis, strain and strain rate, are used in the evaluation of the global and regional functions of the myocardium. Deformation analysis is performed in two ways. One is tissue doppler imaging, the other is two-dimensional speckle tracking or velocity vector imaging (5). The currently preferred method is the tracking of the contraction and relaxation process of the myocardium. The most important advantages of the two-dimensional speckle tracking method are that it is independent of angle and less affected by translational movements. RV longitudinal strain is measured from the apical four chambers.

The interventricular septum affects the calculation of RV longitudinal strain because it is a part of both ventricles.

A study involving 27 patients with severe systolic heart failure (ejection fraction <25%) showed that RV free wall strain was well correlated with myocardial fibrosis detected by histological analysis (6). In a study conducted by Cameli and colleagues, RV GLS has been found to be the strongest predictor for clinical outcomes in patients with heart failure undergoing heart transplantation (7). Similarly, Garcia-Martin and colleagues have shown that RV GLS is a stronger predictor of heart failure development than TAPSE (8). Vizzardi and colleagues have shown that RV GLS is stronger predictors in hospital admission for cardiac death and heart failure than TAPSE and RV FAC. The prognostic superiority of RV GLS can be explained by its more accurate reflection of decreased RV strain and severely impaired RV performance (9). In our study, similarly, it was observed that the values of RV Global strain and RV Free strain were significant (Table 3 and Figure 1).

Hasselberg and colleagues demonstrated that in a population including patients with low ejection fraction heart failure and heart failure with preserved ejection fraction, RV GLS is a good predictor of low functional capacity (peak oxygen consumption <20 ml/kg/min). Unlike our study, the authors did not analyze patients with low ejection fraction of HF separately (10).

**Figure 3:**
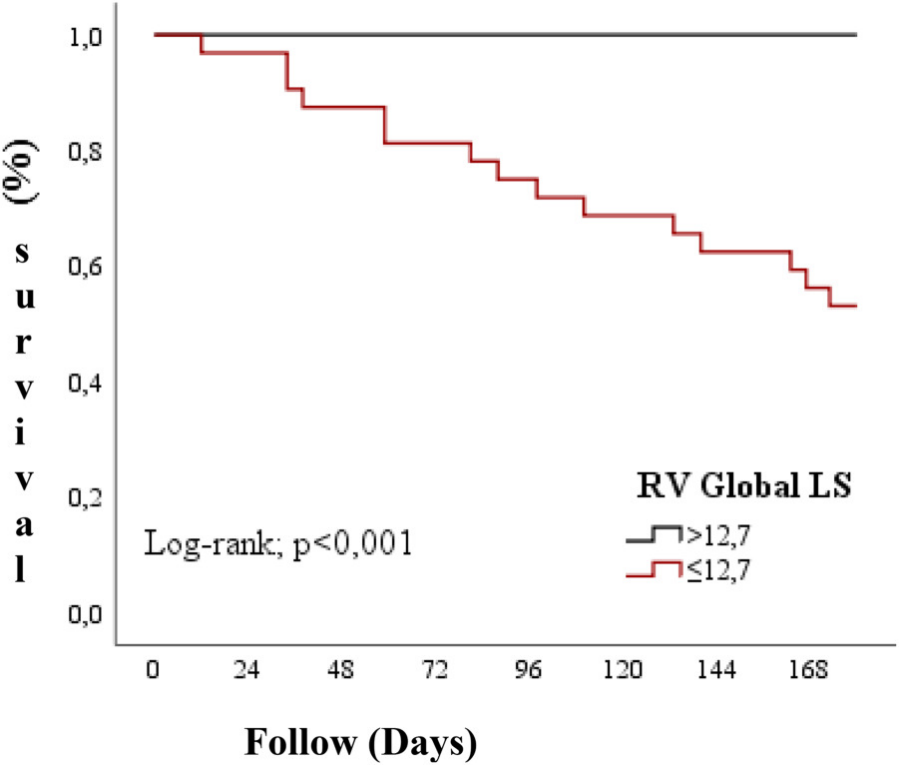
Survival based on RV global LS values.

Guendouz and colleagues compared 108 patients with low ejection fraction to a control group and showed that RV longitudinal strain was strongly associated with primary endpoints (death, emergency heart transplantation, emergency ventricular assist device implantation, or admission for acute heart failure requiring intravenous medication). This relationship was independent of clinical and laboratory variables, including TAPSE, RV diastolic end area, tissue doppler and LV diastolic end diameter. The authors have determined a cut-off value of −21% for RV GLS to differentiate patients with and without primary endpoint after 1 year of follow-up (11). In our study, similar results were obtained with an optimal cut-off value determined by the Youden index for RV Global LS of −12.7% and for RV Free W Strain of −14.6% (Figures 2, 3).

Mateli and colleagues performed echocardiographic examination and right heart catheterization simultaneously on 47 patients referred for heart transplantation due to advanced stage heart failure. The authors found that there is no relationship between RV stroke volume and TAPSE or tricuspid S', but they found a negative correlation between RV free wall strain and RV stroke volume. Furthermore, the prognostic value of RV free wall strain was higher than RV global strain, TAPSE, and tricuspid S' (12).

Another study, which included 332 KY patients followed for 36 ± 26 months, demonstrated that both RV Global longitudinal Strain and RV free wall strain were associated with all-cause mortality or cardiovascular mortality. Furthermore, RV Global longitudinal strain and RV free wall strain were associated with mortality independent of main clinical and echocardiographic variables. The RV global LS and RV free wall strain cut-off values were calculated as −14.0% and −20.6% (13). In our study, age, mitral lateral E E', RV Global LS, and RV Free W Strain were found to be associated with overall survival (Table 2).

The clinical relevance of our findings lies in the fact that RV GLS and RV FWS are simple to measure with standard echocardiographic equipment and provide incremental prognostic value beyond traditional markers like TAPSE or RV FAC. These parameters can assist clinicians in identifying high-risk patients during hospitalization and at discharge, allowing for closer outpatient follow-up, timely therapeutic adjustments, and potentially improved survival outcomes.

In our study, we found that RV GLS and RV Free WS are closely related. Additionally, both measurements were correlated not only with parameters reflecting RV systolic function but also with parameters reflecting LV function. However, in multivariable analysis, both RV GLS and RV Free WS had a similar prognostic role. In our study, it has been clearly shown that RV GLS and RV Free WS are better prognostic predictors than other echocardiographic parameters such as TAPSE, tricuspid S', and FAC. While our study focused on a broader composite outcome, future research should aim to isolate predictors of specific events such as Major Adverse Cardiac Events (MACE), which may provide more targeted insights into the role of RV strain in heart failure prognosis.

## Conclusion

We found that RV GLS and RV Free WS are closely related. Additionally, both measurements were correlated not only with parameters reflecting RV systolic function but also with parameters reflecting LV function. Our study is single-centered and limited by its sample size. Therefore, multicenter studies with larger patient populations are warranted to confirm the prognostic significance of RV GLS and RV Free Wall Strain and evaluate their integration into routine clinical practice for heart failure management.

## Data Availability

The datasets presented in this study can be found in online repositories. The names of the repository/repositories and accession number(s) can be found in the article/Supplementary Material.
